# The functional "KL-VS" variant of *KLOTHO *is not associated with type 2 diabetes in 5028 UK Caucasians

**DOI:** 10.1186/1471-2350-7-51

**Published:** 2006-06-05

**Authors:** Rachel M Freathy, Michael N Weedon, David Melzer, Beverley Shields, Graham A Hitman, Mark Walker, Mark I McCarthy, Andrew T Hattersley, Timothy M Frayling

**Affiliations:** 1 Institute of Biomedical and Clinical Science, Peninsula Medical School, Exeter, UK; 2 Centre for Diabetes and Metabolic Medicine, Barts and The London Queen Mary's School of Medicine and Dentistry, London, UK; 3 School of Clinical Medical Sciences, University of Newcastle upon Tyne, Newcastle upon Tyne, UK; 4 Oxford Centre for Diabetes, Endocrinology and Metabolism, Headington, Oxford, UK

## Abstract

**Background:**

Klotho has an important role in insulin signalling and the development of ageing-like phenotypes in mice. The common functional "KL-VS" variant in the *KLOTHO *(*KL*) gene is associated with longevity in humans but its role in type 2 diabetes is not known. We performed a large case-control and family-based study to test the hypothesis that KL-VS is associated with type 2 diabetes in a UK Caucasian population.

**Methods:**

We genotyped 1793 cases, 1619 controls and 1616 subjects from 509 families for the single nucleotide polymorphism (SNP) F352V (rs9536314) that defines the KL-VS variant. Allele and genotype frequencies were compared between cases and controls. Family-based analysis was used to test for over- or under-transmission of V352 to affected offspring.

**Results:**

Despite good power to detect odds ratios of 1.2, there were no significant associations between alleles or genotypes and type 2 diabetes (V352 allele: odds ratio = 0.96 (0.84–1.09)). Additional analysis of quantitative trait data in 1177 healthy control subjects showed no association of the variant with fasting insulin, glucose, triglycerides, HDL- or LDL-cholesterol (all *P *> 0.05). However, the HDL-cholesterol levels observed across the genotype groups showed a similar, but non-significant, pattern to previously reported data.

**Conclusion:**

This is the first large-scale study to examine the association between common functional variation in *KL *and type 2 diabetes risk. We have found no evidence that the functional KL-VS variant is a risk factor for type 2 diabetes in a large UK Caucasian case-control and family-based study.

## Background

Klotho is a mammalian hormone with an important role in insulin signalling and the development of ageing-like phenotypes. Klotho binds to a cell surface receptor and represses intracellular signals of insulin and insulin-like growth factor-I (IGF-I) [1]. Mice with a defect in *Klotho *expression exhibit phenotypes that are associated with ageing in humans, including short life span, arteriosclerosis and osteoporosis [2]. These mice have reduced blood glucose and insulin levels, as well as increased insulin sensitivity [2,3]. Conversely, over-expression of *Klotho *in mice extends life span and results in insulin resistance [1].

A common variant of the human *KLOTHO *gene (*KL*), is reproducibly associated with longevity [4,5]. The Klotho protein is encoded by a 50-kb gene on chromosome 13q12, which consists of 5 exons [6]. A haplotype, "KL-VS", composed of six single nucleotide polymorphisms (SNPs), spans exon 2 and its flanking sequence and is present in approximately 15% of Caucasians [4]. Two of these SNPs result in amino acid substitutions: F352V (rs9536314) and C370S (rs9527025). "KL-VS" refers to the V and S alleles of these SNPs respectively, and since all six SNPs occur in perfect linkage disequilibrium, a single variant, F352V, can be used to tag the haplotype [4]. There is a lower frequency of VV homozygotes in elderly individuals than in newborns (three independent populations: *P *= 0.05–0.08; combined analysis (*n *= 2416): *P *= 0.0023) [4]. F352V occurs at a completely conserved amino acid, and *in vitro *work shows that KL-VS influences *Klotho *expression [4].

The KL-VS variant has been variably associated with early-onset coronary artery disease (CAD) [7,8] and related phenotypes: reduced HDL-cholesterol, increased systolic blood pressure and stroke [5]. However, these studies are relatively small and require replication.

The role of the *KLOTHO *gene in type 2 diabetes is not known. Given the importance of Klotho in insulin signalling [1-3] and the reduction in insulin production which co-exists with ageing-related disease in Klotho-deficient mice [3], we hypothesised that the F352V polymorphism in *KLOTHO *would be associated with type 2 diabetes in the UK Caucasian population. Here we present the results of a large study, powered to detect odds ratios of ~1.2, which examines the role of this common functional *KLOTHO *variation in type 2 diabetes.

## Methods

### Subjects

The clinical characteristics of study subjects are shown in Table 1 and Supplementary Table 1 [see Additional File 1]. All participants gave their informed consent. Case subjects were unrelated UK Caucasians with type 2 diabetes. Cases were included either if they had type 2 diabetes, as defined by World Health Organisation criteria [9], or if they were being treated with medication for diabetes. Cases were recruited from three sources, as described previously [10,11]: a collection of young-onset subjects (YT2D; age at diagnosis 18–45 years; *n *= 256); probands from type 2 diabetic sibships from the Diabetes UK Warren 2 repository (W2SP; *n *= 499) [12,13]; and a more recent collection of cases with type 2 diabetes from the Diabetes UK Warren 2 repository (W2C; age at diagnosis 35–65 years; not selected for having a family history of diabetes; *n *= 1038).

**Table 1:** Clinical characteristics of subjects in study.

	Case Subjects	Control Subjects	Warren 2 Trios and Duos Probands
*n*	1793	1619	509
Male (%)	58.8	49.4	58.0
Age (years)*	51 (44–58)	32 (29–35)	41 (36–47)
BMI (kg/m^2^)	30.1 (26.9–34.3)	24.8 (22.1–27.9)	32.3 (28.4–37.2)
Treatment (% D/O/I)	11/62/27	-	18/63/19

Continuous data are given as median (interquartile range). Only successfully genotyped subjects are included.

BMI, body mass index; D/O/I, diet/oral hypoglycaemic agents/insulin. No clinical characteristics were available for the ECACC population control subjects, so control age and BMI are for the Exeter Family Study subjects only.

*Age at diagnosis for case subjects; age at study for control subjects.

Population control subjects were unrelated UK Caucasians recruited from two sources: parents from a consecutive birth study (Exeter Family Study of Childhood Health (EFS) [14]; *n *= 1177) with normal (< 6.0 mmol/l) fasting glucose and/or normal HbA_1c _(< 6%, Diabetes Control and Complications Trial-corrected) [13]; and a nationally recruited control sample from the European Collection of Cell Cultures (ECACC) (*n *= 442). Data on serum concentrations of fasting insulin, glucose, triglycerides, HDL- and LDL-cholesterol were available for EFS subjects.

Subjects for the family-based analysis were recruited as part of a Warren 2 cohort from across the UK and were independent of subjects in the case-control analysis. These families consisted of either an affected proband with both parents ("trios"; in this study 17 out of the 395 trios were missing one parent) or an affected proband with one parent and at least one sibling ("duos"; 14 out of the 223 siblings in this study were affected). These families have been described previously [10,11,15].

Cases and families in which the proband had high GAD autoantibody levels (> 99^th ^percentile of the normal population) were excluded from the study. Clinical criteria and/or genetic testing were used to exclude known subtypes of diabetes such as maturity-onset diabetes of the young or mitochondrial inherited diabetes and deafness.

Using the same case-control and family-based samples, associations have recently been shown between the *KCNJ11*, K23 allele, [16] the *HNF4A *P2 promoter haplotype [10] and the *PPARG *P12 allele [17] and type 2 diabetes with odds ratios consistent with other large type 2 diabetes case-control studies and meta-analyses of multiple studies.

### Genotyping and quality control

The polymorphism F352V (rs9536314) was genotyped in all samples. Genotyping was carried out by KBiosciences (Herts, UK) using its own novel system of competitive allele-specific PCR (KASPar). Details of assay design are available from the KBiosciences website [18]. Genotyping accuracy, as determined from the genotype concordance between duplicate samples (11.5% of total), was 100% (0 discrepancies/552 informative duplicates). The genotyping assay success rates were 96.5% for cases, 95.4% for controls and 96.1% for family samples.

The polymorphism C370S (rs9527025) was genotyped in 307 individuals and confirmed to be in perfect linkage disequilibrium with F352V (r^2 ^= 1). As a result, we carried out no further genotyping of this SNP.

Genotypes were in Hardy-Weinberg Equilibrium in cases, controls and family probands (*P *> 0.01), and within each subgroup of cases and controls (*P *> 0.01).

### Statistical analysis

Before combining case and control subgroups, we tested for homogeneity of allele frequencies using Chi-square tests. There were no significant differences among the subgroups (all *P *> 0.2). Odds ratios and *P *values were calculated using Chi-square tests for our case-control analysis. Our sample of 1793 cases and 1619 controls gave us 80% power to detect odds ratios for alleles of 1.20. This power calculation is for a two-tailed *P *value < 0.05, assuming a control V allele frequency of 0.16.

To analyse our family data, we used the FBAT program [19,20], and we confirmed the result using the TDT/sibTDT method [21].

To assess the degree of linkage disequilibrium between the SNPs, we performed a simple correlation analysis using SPSS v. 11.5 for Windows. We used the ANOVA and General Linear Model commands in SPSS to analyse the quantitative trait data available for EFS subjects against genotype. One-way ANOVA was used to test the null hypothesis of no difference in the mean of each trait among all three genotype groups. In addition we used a recessive model to test the hypothesis that VV individuals have lower HDL-cholesterol than FF/FV individuals, since our data for this trait showed a similar (yet non-significant) relationship with genotype to that reported previously [5].

## Results

Genotype and allele frequencies for cases and controls are displayed in Table 2. Genotype and allele frequencies are shown separately for each subgroup of cases and controls in Supplementary Table 2 [see Additional File 2]. There were no significant associations between alleles or genotypes and type 2 diabetes.

**Table 2:** Result of case/control and FBAT analyses for SNP F352V.

Genotype or Allele	Case-Control Analysis				FBAT		
	
	Controls (N = 1619) Number (Frequency)	Cases (N = 1793) Number (Frequency)	Odds Ratio (95% CI)	*P*	Observed Transmissions (V allele)	Expected Transmissions (V allele)	*P*
FF	1158 (0.72)	1296 (0.72)					
FV	409 (0.25)	447 (0.25)	0.98 (0.84–1.14)	0.74			
VV	52 (0.03)	50 (0.03)	0.86 (0.58–1.28)		136	132	0.57
			
F	2725 (0.84)	3039 (0.85)					
V	513 (0.16)	547 (0.15)	0.96 (0.84–1.09)	0.50			

*P *values for the case-control analysis (two-tailed) were calculated using Chi-square tests with a 3 × 2 contingency table (genotypes; 2 d.f.) or a 2 × 2 contingency table (alleles; 1 d.f.). Odds ratios were calculated relative to genotype FF or allele F. CI, confidence interval.

The results of the FBAT analysis are also presented in Table 2. There was no significant over- or under-transmission of V352 to the affected offspring. A TDT/sibTDT analysis confirmed this result (*P *= 0.62).

Analysis of quantitative trait data in the EFS subjects showed no evidence for an effect of genotype on fasting insulin, fasting glucose, Homeostasis Model Assessment of Insulin Sensitivity (HOMA %S), fasting triglycerides, HDL-cholesterol or LDL-cholesterol (all *P *> 0.1). These results are shown in Table 3, corrected for sex. Females were pregnant (28 weeks' gestation) when biochemical data were collected. Analysis of the sexes separately did not yield any significant associations. However, there was weak evidence that VV males had lower HDL-cholesterol levels than FF/FV males (*P *= 0.07; recessive model). Additional correction for age and BMI made little difference to most results, but increased the evidence that VV males had lower HDL-cholesterol (*P *= 0.05; recessive model).

**Table 3:** Quantitative traits of EFS parents (*n *= 1177), stratified by F352V genotype.

	FF (95% CI)	FV (95% CI)	VV (95% CI)	*P*
Age (years)	31.9 (31.5–32.2)	32.1 (31.5–32.8)	31.3 (29.5–33.2)	0.68
BMI (kg/m^2^)	25.0 (24.7–25.2)	24.9 (24.4–25.4)	24.9 (23.7–26.3)	0.96
Fasting insulin (pmol/l)	58.2 (56.1–60.3)	60.0 (56.5–63.8)	59.3 (49.7–70.8)	0.68
Fasting glucose (mmol/l)	4.51 (4.48–4.53)	4.52 (4.48–4.56)	4.52 (4.39–4.66)	0.88
HOMA %S	81.7 (78.7–84.5)	78.0 (73.3–82.8)	79.3 (66.5–94.4)	0.43
Fasting triglycerides (mmol/l)	1.61 (1.56–1.66)	1.67 (1.58–1.75)	1.81 (1.56–2.10)	0.19
LDL-cholesterol (mmol/l)	3.21 (3.14–3.28)	3.22 (3.11–3.34)	3.18 (2.88–3.52)	0.96
HDL-cholesterol (mmol/l)	1.63 (1.61–1.66)	1.64 (1.60–1.69)	1.56 (1.44–1.69)	0.45
HDL-cholesterol (mmol/l)*	1.64 (1.61–1.66)		1.56 (1.44–1.69)	0.23

Data are given as mean (95% confidence interval). For traits other than age, results are corrected for sex. For the serum fasting glucose analysis, only values < 6.0 mmol/l were included. All variables apart from age were log-transformed before analysis.

*Recessive model. CI, confidence interval; BMI, body mass index; HOMA %S, Homeostasis Model Assessment of Insulin Sensitivity.

## Discussion

*KLOTHO *is a type 2 diabetes candidate gene due to its importance in insulin signalling, as demonstrated by murine models, and its potential role in longevity [1-5]. We hypothesised that functional variant V352 (KL-VS) would be associated with type 2 diabetes. Our case-control and family-based study results gave no support for this, despite good power to detect odds ratios of 1.2. Our study is the first well-powered study of this important variant in type 2 diabetes. Two previous studies have examined KL-VS in relation to diabetes (approximately 60 affected individuals; approximately 350 unaffected) in cohorts ascertained for longevity [5] and CAD [7] and found no association. Whilst we have not captured all common variation across *KL *and cannot rule out the effects of rare variants, we have tested a variant which is functional and reproducibly associated with longevity. We can exclude the KL-VS variant from having all but a minor effect (OR < 1.09 relative to the F allele) on type 2 diabetes susceptibility.

There is suggestive evidence that the KL-VS variant is associated with a higher risk of early-onset CAD [7] and with cardiovascular disease risk factors such as HDL-cholesterol [5]. Together with the longevity association data, these findings imply a possible role in susceptibility to metabolic syndrome. However, the studies are relatively small. Analysis in our study of quantitative trait data from 1177 healthy individuals showed no significant association of KL-VS with HOMA %S, fasting insulin, glucose, triglycerides, HDL- or LDL-cholesterol. We were unable to exclude individuals who were taking lipid-lowering medication from this analysis, but since > 92% of these subjects were aged below 40 years, this proportion of subjects is likely to be negligible. Whilst these subjects are younger on average than those analysed previously [5,7], we have analysed a sample that is over twice as large and found no evidence for association of KL-VS with these phenotypes in the group as a whole. Thus it is possible that the previous association with HDL-cholesterol represents a false positive. We note, however, that our HDL-cholesterol result shows a similar pattern to previously reported data [5] and is approaching significance for an association of the VV genotype with lower HDL-cholesterol in males, but not in pregnant females. We therefore cannot rule out an effect and conclude that further large-scale studies will be required to address this.

## Conclusion

This is the first adequately powered study to examine the association between common functional variation in *KLOTHO *and type 2 diabetes. We have examined a polymorphism which has been reproducibly associated with human longevity, but found no evidence that it is involved in the genetic susceptibility to type 2 diabetes in our large case-control and family-based study.

## Abbreviations

CAD, coronary artery disease; ECACC, European Collection of Cell Cultures; EFS Exeter Family Study; FBAT, Family-based association test; *KL*, *KLOTHO*; SNP, single nucleotide polymorphism; TDT, transmission-disequilibrium test; W2C, Warren 2 Cases; W2SP, Warren 2 sib-pair probands; W2TDP, Warren 2 trios and duos probands; YT2D, young-onset type 2 diabetes.

## Competing interests

The authors declare that they have no competing interests.

## Authors' contributions

RMF carried out the data analysis and drafted the manuscript. MNW supervised the data analysis and, together with BS, was responsible for database management. GAH, MW, MIM and ATH conceived and designed the Warren 2 studies. TMF and DM conceived and designed this study. TMF co-ordinated the study and supervised the redrafting of the manuscript. All authors read and approved the final manuscript.

## Pre-publication history

The pre-publication history for this paper can be accessed here:



## Supplementary Material

Additional File 1 **Supplementary **Table 1. **Clinical characteristics of subjects by study group**. Table of supplementary data showing clinical characteristics of the individual study groups involved in this study. Click here for file

Additional File 2 **Supplementary **Table 2. **Genotype and allele numbers (and frequencies) by study**. Table of supplementary data showing detailed genotype information on the individual study groups involved in this study. Click here for file
