# Supplementary material for: The functional "KL-VS" variant of KLOTHO is not associated with type 2 diabetes in 5028 UK Caucasians
Source: BMC Med Genet. 2006 Jun 5;7:51. doi: 10.1186/1471-2350-7-51 (PMC1534014; doi:10.1186/1471-2350-7-51)
Supplement: Additional File 1 — Supplementary Table 1. Clinical characteristics of subjects by study group. Table of supplementary data showing clinical characteristics of the individual study groups involved in this study. [file 1471-2350-7-51-S1.doc]

**Supplementary Table 1. Clinical characteristics of subjects by study group.**

|  | Case Subjects | | | Control Subjects | | Family-Based Subjects |
| --- | --- | --- | --- | --- | --- | --- |
|  | W2C | W2SP | YT2D | EFS Parents | ECACC  Human Random UK Controls | W2TDP |
| *n* | 1038 | 499 | 256 | 1177 | 442 | 509 |
| Male (%) | 62.0 | 53.9 | 55.5 | 48.9 | 51.6 | 58.0 |
| Age (years)* | 52 (45-57) | 57 (50-62) | 40 (36-44) | 32 (29-35) | NA | 41 (36-47) |
| BMI (kg/m^2^) | 30.9 (27.4-35.1) | 28.1 (25.3-31.4) | 31.9 (28.1-36.2) | 24.8 (22.1-27.9) | NA | 32.3 (28.4-37.2) |
| Treatment (% D/O/I) | 9/65/26 | 18/68/14 | 9/53/38 | - | - | 18/63/19 |

Continuous data are given as median (interquartile range). Only successfully genotyped subjects are included.

BMI, body mass index; D/O/I, diet/oral hypoglycaemic agents/insulin; ECACC, European Collection of Cell Cultures; EFS Exeter Family Study; NA, Not available; W2C, Warren 2 Cases; W2SP, Warren 2 sib-pair probands; W2TDP, Warren 2 trios and duos probands; YT2D, young-onset type 2 diabetes.

*Age at diagnosis for case subjects; age at study for control subjects.
