# Supplementary material for: The functional "KL-VS" variant of KLOTHO is not associated with type 2 diabetes in 5028 UK Caucasians
Source: BMC Med Genet. 2006 Jun 5;7:51. doi: 10.1186/1471-2350-7-51 (PMC1534014; doi:10.1186/1471-2350-7-51)
Supplement: Additional File 2 — Supplementary Table 2. Genotype and allele numbers (and frequencies) by study. Table of supplementary data showing detailed genotype information on the individual study groups involved in this study. [file 1471-2350-7-51-S2.doc]

**Supplementary Table 2. Genotype and allele numbers (and frequencies) by study.**

|  | **Case subjects** | | | **Control subjects** | | **Families** |
| --- | --- | --- | --- | --- | --- | --- |
|  | W2C | W2SP | YT2D | EFS Parents | ECACC Human Random Controls | W2TDP |
| **F352V** |  |  |  |  |  |  |
| **FF** | 744 (0.72) | 359 (0.72) | 193 (0.75) | 847 (0.72) | 311 (0.70) | 352 (0.69) |
| **FV** | 269 (0.26) | 121 (0.24) | 57 (0.22) | 294 (0.25) | 115 (0.26) | 144 (0.28) |
| **VV** | 25 (0.02) | 19 (0.04) | 6 (0.02) | 36 (0.03) | 16 (0.04) | 13 (0.02) |
|  |  |  |  |  |  |  |
| **F** | 1757 (0.85) | 839 (0.84) | 443 (0.87) | 1988 (0.84) | 737 (0.83) | 848 (0.83) |
| **V** | 319 (0.15) | 159 (0.16) | 69 (0.13) | 366 (0.16) | 147 (0.17) | 170 (0.17) |

ECACC, European Collection of Cell Cultures; EFS Exeter Family Study; W2C, Warren 2 Cases; W2SP, Warren 2 sib-pair probands; W2TDP, Warren 2 trios and duos probands; YT2D, young-onset type 2 diabetes.
